# A Rare Post-traumatic Tibial Tubercle Avulsion Combined With a Complete Section of the Patellar Tendon in a Young Patient: Insight Into Surgical Management

**DOI:** 10.7759/cureus.100600

**Published:** 2026-01-02

**Authors:** Hafid Talha

**Affiliations:** 1 Department of Pediatric Surgery, Moulay Ali Cherif Regional Hospital Center, Errachidia, MAR; 2 Laboratory of Health Sciences, Faculty of Medicine and Pharmacy, Moulay Ismail University, Errachidia, MAR

**Keywords:** knee trauma, patellar tendon rupture, staples fixation, tibial tubercle avulsion, traffic accident

## Abstract

Avulsion of the tibial tubercle with patellar tendon section in children is a rare but serious injury. It typically occurs after acute trauma, such as a forceful take-off or sudden jump in a young person nearing skeletal maturity. Diagnosis is made on plain radiographs, and displaced forms require surgical treatment to realign and fix the bone fragment and repair the tendon. We report here the case of a 15-year-old adolescent involved in a road traffic accident with a direct impact to the left knee. Standard radiographs showed a fracture of the tibial tubercle. Intraoperative exploration revealed a combined tibial tubercle avulsion and a complete section of the patellar tendon at its insertion. Fixation of the displaced tubercle and the patellar tendon using two staples was performed, with a favorable postoperative course and good functional outcome. Later, at the six-month follow-up, examination of the left knee demonstrated a full, pain-free range of motion, with complete active extension and full flexion.

## Introduction

Tibial tubercle avulsion is an uncommon lesion, typically seen in skeletally immature patients whose tibial physis remains open [1]. Concomitant patellar tendon rupture in the setting of a tibial tubercle avulsion fracture is exceptionally uncommon [2]. Several mechanisms have been proposed, most often involving excessive force applied to the tibial tubercle, such as landing from a jump or a forceful quadriceps contraction [3]. The injury in our patient resulted from a direct impact to the left knee during a traffic accident. These lesions are serious and require prompt diagnosis and surgical management [4]. Because of their rarity, there is no standardized surgical technique, although open reduction and internal fixation are generally favored to treat the avulsion and are associated with good clinical and functional outcomes [5], taking into account pre-injury knee function and activity level [3]. Through this case report, we aim to highlight the surgical technique used in our patient, in which staples were employed for tibial tubercle and patellar tendon fixation. The postoperative course was very favorable, with the patient regaining a full range of motion and complete restoration of extensor strength compared with the contralateral knee.

## Case presentation

**Figure 1:**
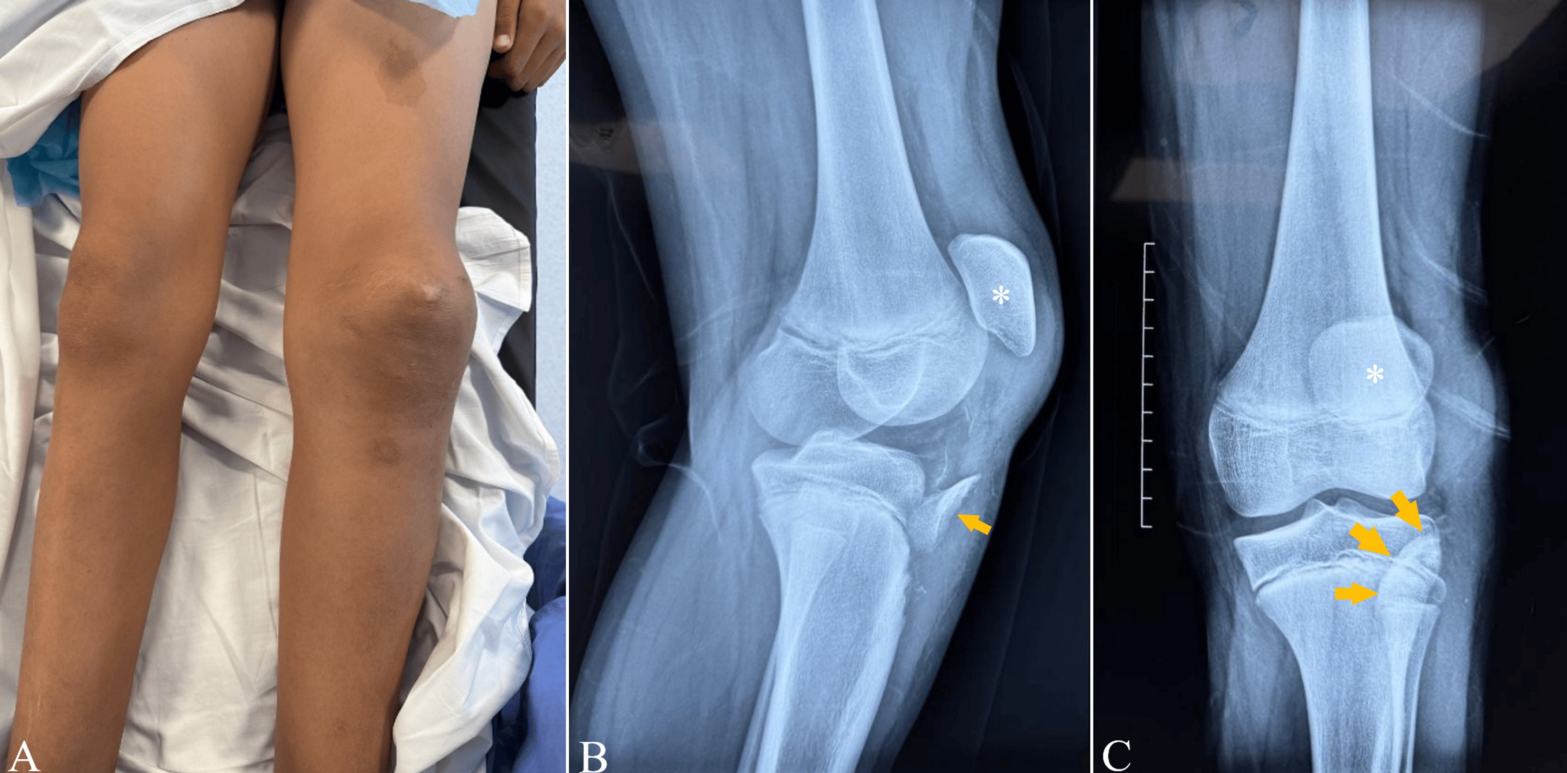
Clinical appearance of the left knee at admission (A). Preoperative X-rays of the left knee showing an avulsion fracture of the tibial tubercle (arrows) with patella alta (asterisk) on the lateral (B) and anteroposterior (C) views

This is the case of a 15-year-old adolescent who presented with left knee trauma following a scooter accident, with a direct impact to the anterior aspect of the left knee. He reported immediate pain and complete loss of function of the left knee. On admission, clinical examination revealed a swollen left knee with complete functional impairment (Figure 1A). Active extension was impossible, and passive flexion of the knee could not be achieved due to pain. Standard radiographs of the affected knee demonstrated a displaced fracture of the tibial tubercle, associated with proximal migration and lateral displacement of the patella (Figure 1B-1C).

**Figure 2:**
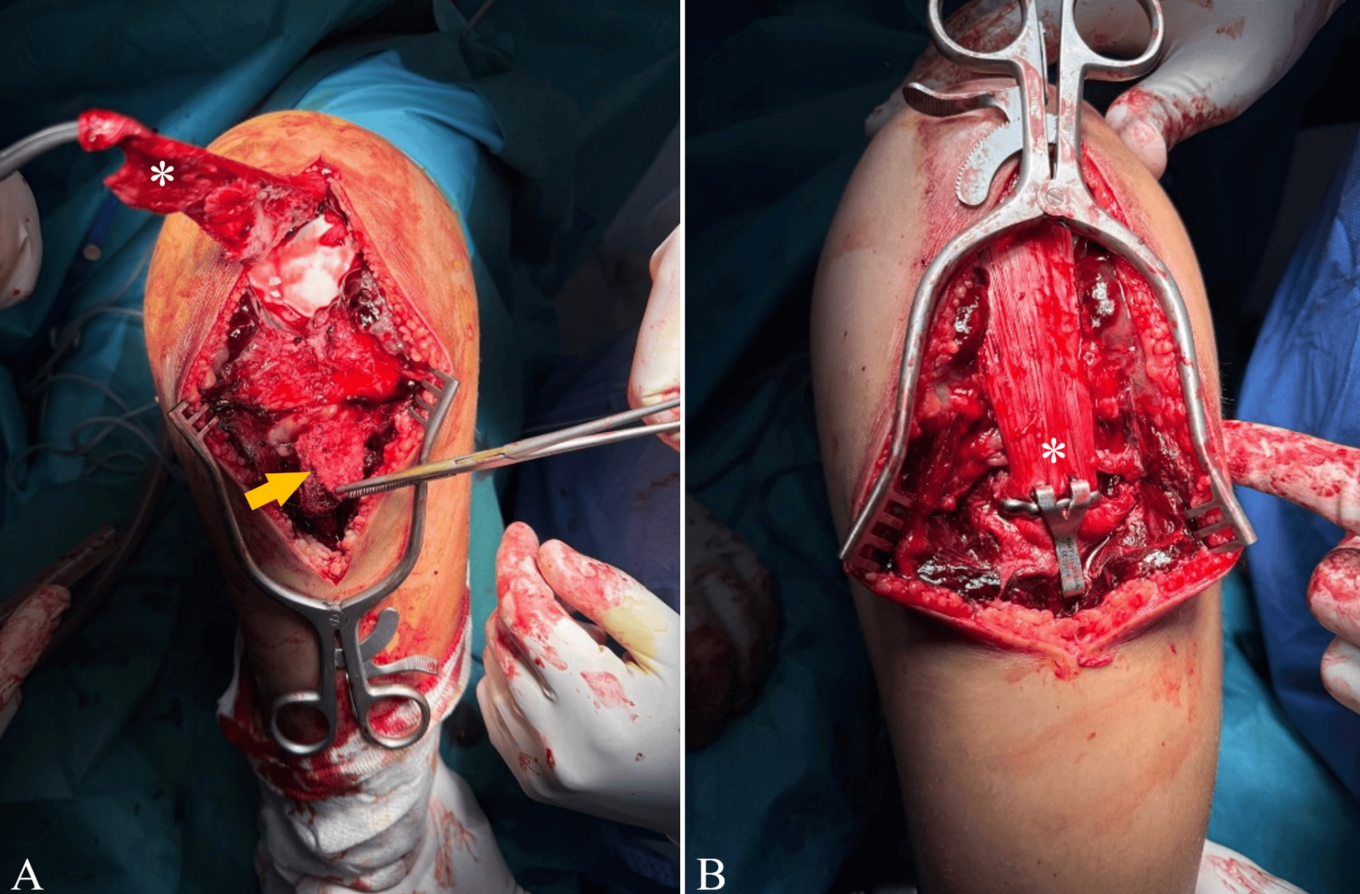
Operative view of the detached patellar tendon (asterisks) and the tibial tubercle (arrow) before (A) and after fixation using a Blount staple (B)

Surgical management was strongly recommended to reduce the displaced tibial tubercle and restore the integrity of the knee extensor mechanism. The patient was taken to the operating room and underwent surgery under spinal anesthesia. An anterior approach to the knee was performed, centered over the patella and the tibial tubercle. Evacuation of the peri-fracture hematoma was carried out. Intraoperative exploration revealed avulsion of the tibial tubercle, complete detachment of the patellar tendon from its tibial insertion, proximal migration of the patella, and disruption of both the medial and lateral retinacula. Reattachment of the patellar tendon was performed using a standard Blount staple, and fixation of the tibial tubercle was achieved with another staple (Figure 2A-2B). The medial and lateral retinacula were repaired, and layered wound closure was performed over a surgical drain. The knee was immobilized in extension in a Zimmer knee splint (rigid, removable knee immobilizer).

**Figure 3:**
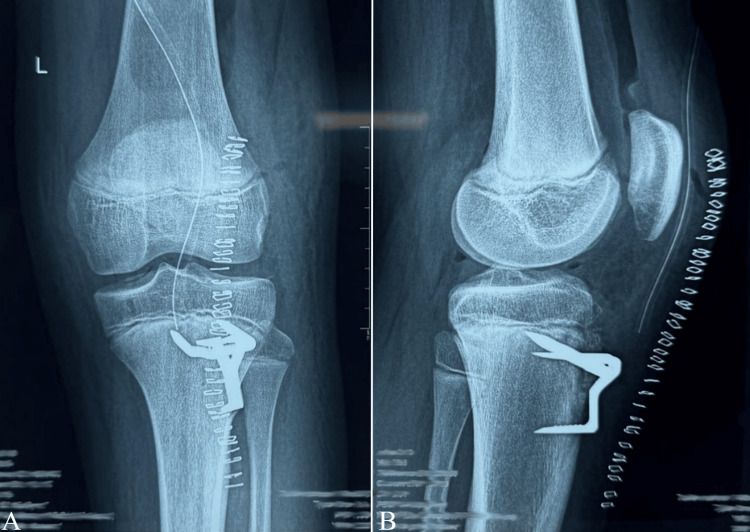
Postoperative radiographs of the left knee one day after the surgical fixation of the tibial tubercle using staples, in anteroposterior (A) and lateral (B) views

**Figure 4:**
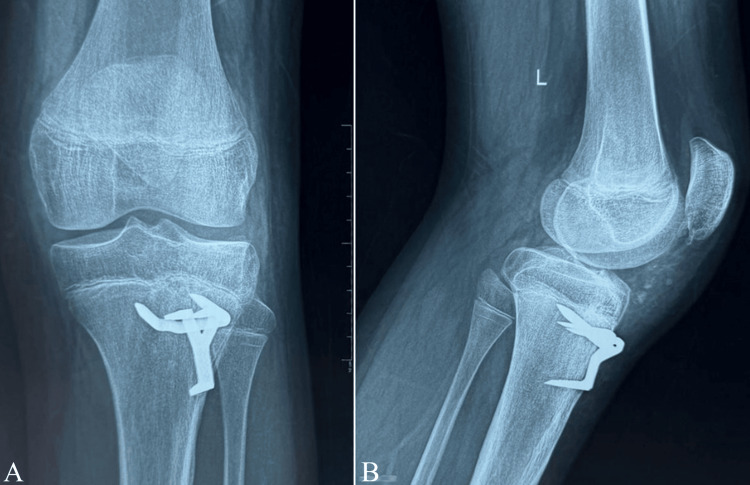
Postoperative radiographs of the left knee six months after surgical fixation and subsequent knee immobilization, in anteroposterior (A) and lateral (B) views

Postoperatively, the knee was immobilized in the Zimmer splint for three weeks. Passive mobilization of the patella was initiated on postoperative day 10. Protected weight-bearing with the knee locked in extension in the splint was allowed from postoperative day 30, and full weight-bearing without the splint was authorized at postoperative day 45. X-rays performed on postoperative day 1 (Figure 3A-3B) and at six months postoperatively (Figure 4A-4B) showed that the staples were in the correct position. Our patient presented with a type Ib fracture of the tibial tubercle.

**Figure 5:**
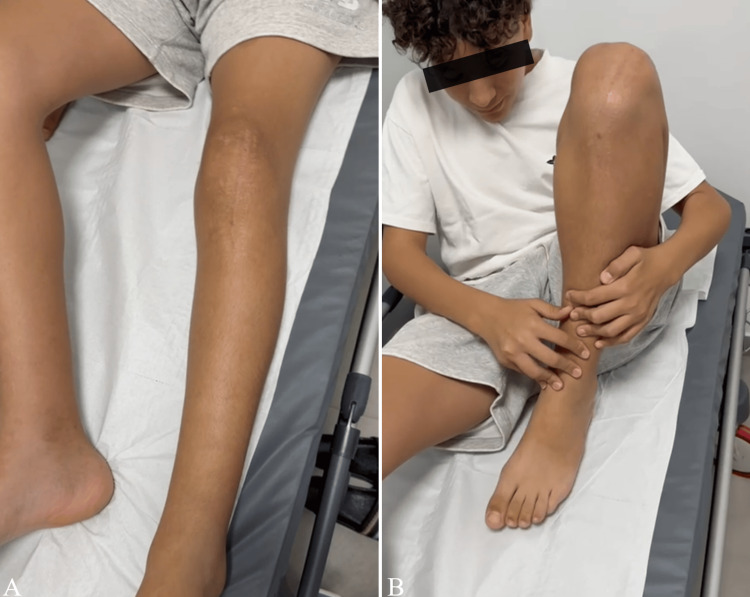
Six-month postoperative clinical evaluation of the left knee demonstrating full range of motion, with complete active extension (A) and full flexion (B)

At the six-month follow-up, physical examination of the left knee showed a full, pain-free range of motion, with complete active extension and full flexion of the knee (Figure 5A-5B).

## Discussion

Tibial tubercle avulsion fracture is a rare type of proximal tibial epiphyseal injury, accounting for approximately 1% of all physeal fractures [6]. Tubercle avulsion is considerably more common in adolescents than in adults because of the relative weakness of the open physis [7]. These injuries typically occur in young males, most often during sports activities [8]. The usual mechanism involves rapid knee flexion against a forcefully contracted quadriceps, sprinting or kicking, or oppositional forces [9]. They may also result from a direct blow to the anterior knee, as in our case. The tibial tubercle and the patellar tendon insertion represent the last region of the proximal tibial physis to close, usually between 10 and 15 years of age in males, which further contributes to vulnerability at this site [8]. Certain conditions, such as Osgood-Schlatter disease, connective tissue disorders, and osteogenesis imperfecta, have also been reported to increase the risk of tibial tubercle injury [3].

Clinically, patients often describe a "snapping" at the time of injury, followed by the inability to actively achieve full knee extension, along with pain, and knee effusion [3]. Physical examination typically reveals a palpable defect inferior to the patella, decreased range of motion, and inability to perform or maintain active knee extension [10]. Imaging is essential to confirm the diagnosis and guide management. CT is useful for the detailed assessment of the articular surface and for preoperative planning, whereas MRI is indicated when there is concern for intra-articular extension of the fracture [4].

Three main classification systems have been used to describe tibial tubercle fractures: the Watson-Jones classification, which was the first to characterize these injuries [11], as well as the Ogden and Salter-Harris classifications. According to the Ogden classification, tibial tubercle fractures are grouped into five types. Type I involves a fracture confined to the secondary ossification center at the insertion of the patellar tendon. In type II, the fracture line extends proximally between the primary and secondary ossification centers. Type III corresponds to a coronally oriented fracture that extends posteriorly through the primary ossification center. Type IV injuries traverse the entire proximal tibial physis. Type V refers to a periosteal sleeve avulsion in which the extensor mechanism is stripped from the secondary ossification center. Each type is further designated A if nondisplaced and B if displaced [12]. According to the Ogden classification, our patient had a type Ib fracture. Although there are no formal guidelines defining the optimal treatment strategy, the strong pull of the patellar tendon means that displaced fractures managed nonoperatively have a high risk of persistent displacement and impaired fracture healing [13]. Internal fixation uses partially threaded cancellous screws in skeletally mature patients, while Kirschner wires are used in skeletally immature patients [14]. These lesions have been treated using a variety of fixation methods, with the choice largely depending on surgeon preference [7].

In our case, the use of staples was chosen to achieve solid fixation of the distal patellar tendon, and a second staple was used to reattach the tibial tubercle to its original position. Postoperatively, there is still no consensus regarding the optimal timing for weight-bearing and range of motion exercises [3]. In our protocol, the patient was allowed protected weight-bearing with the knee locked in extension in a splint starting one month after surgery, followed by full weight-bearing without the splint two weeks later.

## Conclusions

Tibial tubercle avulsion fractures associated with complete patellar tendon rupture are exceptionally rare and should be suspected in adolescents presenting with acute anterior knee trauma, loss of active extension, and radiographic evidence of tibial tubercle displacement. Early recognition and prompt surgical management are essential to restore the integrity of the extensor mechanism and to prevent long-term functional impairment. This case illustrates that staples fixation of both the tibial tubercle and the patellar tendon insertion can provide stable fixation and allow for a structured rehabilitation protocol, leading to excellent clinical and radiological outcomes.
